# The sensitivity outcome index system for home care of elderly liver transplant patients was developed based on the Omaha problem classification system

**DOI:** 10.1186/s12911-024-02617-w

**Published:** 2024-07-25

**Authors:** Bin Wang, Xia Huang, Guofang Liu, Taohua Zheng, Hui Lin, Yue Qiao, Wenjuan Sun

**Affiliations:** The Affiliated Hospital of Qingdao University, Qingdao City, 266071 Shandong Province China

**Keywords:** Elderly liver transplantation, Omaha problem classification system, Home care, Sensitivity outcome index, Delphi process

## Abstract

**Objective:**

Based on the Omaha problem classification system, a sensitivity outcome index system for home nursing of elderly liver transplant patients was established.

**Methods:**

Through a comprehensive literature review and rigorous application of the Delphi method, a panel of 20 experts completed two rounds of effective letter consultation to obtain expert consensus opinions. The contents of indicators were determined based on this process, and the analytic hierarchy process was employed to confirm the weightage assigned to each indicator. Consequently, we established a sensitivity outcome index system for home care in elderly liver transplant patients.

**Results:**

The effective recovery rate of the questionnaire in two rounds of expert consultation was 100%, and the proportion of experts who gave opinions was 55% and 15%, respectively, indicating that the experts were highly active. The expert authority coefficients were calculated as 0.904 and 0.905, respectively, indicating a high degree of expert authority. In the second round, Kendall’s coordination coefficients for primary, secondary, and tertiary indicators were determined to be 0.419, 0.418, and 0.394 (*P* < 0.001), indicating that expert opinions tended to be consistent. Finally, we established a comprehensive sensitivity outcome index system comprising 4 first-level indexes, 20 s-level indexes, and 72 third-level indexes specifically designed for elderly liver transplantation patients.

**Conclusion:**

The sensitivity outcome index system of home nursing for elderly liver transplant patients can provide theoretical basis for nursing staff to build accurate individualized continuous nursing model.

**Supplementary Information:**

The online version contains supplementary material available at 10.1186/s12911-024-02617-w.

## Introduction

The primary therapeutic approach for end-stage organ failure is transplantation [1]. With the advancement of transplant technology and the increasing elderly population, advanced age is no longer considered a contraindication for liver transplantation. In the 2013 Guidelines for the Evaluation of adult liver transplant recipients, both the American Liver Association and Transplantation Society stated that individuals aged ≥ 70 years are suitable candidates for liver transplantation [2]. However, as the number of procedures and survival rates among elderly liver transplant patients continue to rise, there are also an array of nursing challenges.

It has been reported that elderly patients have very high demands for nursing issues such as drugs, diet and self-monitoring during the home recovery period after surgery, reaching 97.4%, 94.7% and 81.6% respectively [3]. Nursing issues are expressed through a series of specific assessment indicators, enabling the most comprehensive assessment of patient outcomes. However, current sensitivity indicators for liver transplantation in China primarily focus on evaluating the quality of hospitalization, inclined structure and process control. Lacking patient outcome-oriented nursing sensitivity outcome indicators, especially in elderly patients, has not been addressed [4]. The Omaha Problem Classification System encompasses 42 nursing problems and thousands of evaluation indicators across four domains: physiological, psychosocial, health-related behavior, and environment. This system has been extensively utilized to evaluate nursing sensitivity outcomes in various conditions such as diabetes [5], inflammatory bowel disease [6], and bladder cancer after surgery [7], achieving positive results. Therefore, this study adopts the Omaha Question Classification System [8] as its framework and applies the Delphi method [9] to establish a nursing sensitivity outcome index system tailored for elderly patients undergoing home recovery after liver transplantation—providing nurses with a theoretical foundation for implementing an accurate group continuous nursing model.

## Method

### Set up a research group

The research team was established, comprising specialist nurses, head nurses, department directors, outpatient nurses, and researchers. Their primary responsibility involved determining the selection of topics, formulating indicators, selecting experts, collating data, and conducting analysis.

### Develop a sensitivity outcome index system for home nursing of elderly liver transplant patients

In this paper, “Omaha problem classification system”, “elderly liver transplantation”, “home care”, “extended care”, “system construction”, “nursing sensitivity outcome indicators”, “quality evaluation/quality improvement/quality management” were used as the search terms. The search was conducted in Chinese databases (such as CNKI, Wanfang, Weipu) and English databases (such as Pubmed, Sciencedirect, Springer). The search time limit: from the establishment of the database to November 1, 2023.

After sorting and screening literature and discussion by the research group, a draft outcome index system for elderly liver transplant patients’ home care sensitivity based on the Omaha problem classification system was formed. Five experts were invited to participate in a pre-letter consultation. The selection criteria for the inclusion of these experts were as follows: an age range of 47.75 ± 3.34 years old, possessing a professional title of intermediate or above, and being actively involved in liver transplantation nursing more than ten years. After consolidating the expert opinions, a formal letter questionnaire was developed. This questionnaire comprised three sections: (1) An expert letter questionnaire that utilized the Likert 5-level scoring method to assess the importance of indicators. (2) General information about the experts, including their professional titles, ages, education levels, and years of experience. (3) The basis for expert judgment and familiarity with the index questionnaire. (The expert letter questionnaire is detailed in Annex I.)

### Determine the expert for correspondence consultation

A total of 20 experts in China from tertiary hospitals and universities were selected. The pre-set inclusion criteria were as follows: (1) medical or nursing education experts from universities who willingly accepted letter inquiries and committed to continuous participation in the project. (2) possessing intermediate or higher professional and technical titles. (3) having more than 10 years of experience in liver transplantation nursing or being specialist nurses in liver transplantation. (4) holding a Bachelor’s degree or higher.

### Implement expert letter consultation

Our study carried out two rounds of expert consultation from February to April 2023. The paper or electronic questionnaires were distributed by the same researcher to experts, and the interval between each round of survey was two weeks. Likert 5-point scoring method was also used to evaluate the importance of each item. The questionnaire was equipped with a modification column for experts to propose modification, addition and deletion. After two rounds of letter consultation, the expert opinions were basically reached. According to the expert opinions, the indicators were included to meet the criteria of the mean value of importance assignment ≥ 3.50 and the coefficient of variation < 0.25 [10]. After the collective review of the research team, the sensitivity outcome index system of home care for elderly patients with liver transplantation was confirmed.

### Data processing

Using SPSS 22.0 statistical software, data entry, analysis, and finalization were conducted. Count data were presented as frequency and percentage. Measurement data were expressed as mean ± standard deviation and coefficient of variation. The recovery rate of questionnaires and the submission rate of opinions served as indicators for expert positivity coefficient determination. The authority coefficient (Cr) of experts was calculated using the judgment coefficient (Ca) and familiarity coefficient (Cs). The degree of agreement among expert opinions was assessed through the coefficient of variation and Kendall coordination coefficient W.

## Results

### Expert situation

A total of 20 experts from 9 cities, including Qingdao, Shanghai, Guangzhou, Tianjin, Beijing, Guiyang, Xi’an, Wuhan and Hangzhou successfully completed two rounds of effective letter consultation. Among them were 9 liver transplant specialist nurses or professional committee members (45.0%), 5 liver transplant nursing managers (25.0%), 3 liver transplant surgeon experts (15.0%) and 3 liver transplant nursing education experts (15.0%). The average age of the experts was (43.25 ± 2.20) years old. Among them, the working years of liver transplantation were (13.3 ± 2.20) years, 15 experts (75.0%) had associate senior title or above, and 5 experts (25.0%) had intermediate title. There were 9 bachelor degree holders (45.0%), 6 master degree holders (30.0%) and 5 doctor degree holders (25.0%).

### Degree of expert activeness

The effective recovery rate of the two rounds of correspondence questionnaires was 100%, and constructive suggestions were put forward by experts in each round, indicating that experts were highly motivated, as shown in Table 1.

**Table 1:** The degree of expert activeness

Round	Number of questionnaires issued	Number of questionnaires collected	Effective recovery rate (%)	Opinion submission rate (%)
Round 1	20	20	100%	55%
Round 2	20	20	100%	15%

### Degree of expert authority

As shown in Table 2, two rounds of correspondence of expert authority coefficient (Cr) > 0.80. This suggests that the study possesses a significant level of expert authority and yields credible research findings.

**Table 2:** Expert authority degree

Round	Judgment (Ca)	Familiarity (Cs)	Authority (Cr)
Round 1	0.943	0.865	0.904
Round 2	0.945	0.864	0.905

### Expert opinion coordination degree

The Kendall coordination coefficient W ranges from 0 to 1, and the greater the W, the better the degree of expert coordination [11]. As shown in Table 3, the significance test of the coordination coefficient of the two rounds of letter consultation in our study showed statistical significance, indicating that the expert opinions were coordinated and reliable (Table 4).

**Table 3:** Expert opinion coordination degree and significance test

Index		Round 1				Round 2			
		Coordination coefficient(*W*)	*χ* ^*2*^	Freedom degree(*df*)	*P*	Coordination coefficient (*W*)	*χ* ^2^	Freedom degree (*df*)	*P*
Primary index		0.314	18.828	3	<0.001	0.419	25.163	3	<0.001
Secondary index		0.407	154.511	19	<0.001	0.418	158.662	19	<0.001
Three-level index		0.384	544.848	71	<0.001	0.394	559.995	71	<0.001

### Expert letter inquiry results

**Table 4:** The first and second round of expert letter consultation revised the indicators

Indicators	Reasons for revision
Delete second-level indicators and consists of three-level indicators	
Sadness	“Mental health” includes “sadness”, and “sadness” is modified to the three-level indicator “sadness/depression” under it.
Growth and development	The indicator is removed because the elder had passed the age of growth and development
Pregnancy	The indicator is removed because the elder had reached a pregnancy age, married with children, mostly be deleted
Caregiving/parenting	Repeat the contents of “family care/emotional support”; Most elderly people belong to the care population, so this indicator was removed.
Modify the second-level indicators	
Changed the “contagion/infection” indicator to “infection”.	The issue of contagion among subjects is rarely addressed
The second-level indicators and their subordinate third-level indicators were added	
Knowledge	The elderly have memory decline and slow acceptance ability. “Knowledge” and its three-level indicators should be added to evaluate the patients’ mastery of relevant knowledge
Describe the postoperative dietary precautions	
Describe the precautions for postoperative rehabilitation exercise	
Describe the precautions for postoperative monitoring	
Describe the precautions for postoperative medication management	
Modify the second-level indicators	
The “skin pain” was revised to “skin numbness”, with two three-level indicators of “skin numbness at other parts” and “skin scar numbness at the surgical incision’	During the home operation, the skin of the incision has healed well, and pain rarely occurred. However, due to peripheral nerve injury in the surgical area and partial capillary blood supply disorder, skin numbness and decreased sensation may occur.
Removed the three-level indicators	
Dyspepsia	Dyspepsia mainly manifests as “nausea/vomiting/abdominal distension”, which is more specific.
Follow-up visits were not performed as required	With “extended did not receive medical care” repeat, so delete it.
Modify the three-level indicators	
The “electrolyte disorder” changed to “laboratory index disorder”	After surgery, patients should not only pay attention to electrolytes such as sodium, potassium and calcium, but also pay attention to indicators such as liver function and renal function.

After two rounds of expert consultation and group discussion, 4 first-level indicators, 20 s-level indicators and 72 third-level indicators were finally established. The analytic hierarchy process was used to determine the weight and combined weight of each index, as shown in Table 5.

**Table 5:** Results of expert letter inquiry on evaluation index system of nursing problems of liver transplant patients at home

Pointer code	Importance assignment	Coefficient of variation	Weight	Combined weight		
**1 Environmental field**	5.00 ± 0.00	0.00	0.250	-		
**1.1 Income**	4.75 ± 0.45	0.09	0.074	0.019		
1.1.1 Low/no income	4.25 ± 0.72	0.17	0.009	0.000		
1.1.2 No health insurance 1.1.3 Income does not cover medical expenses	4.75 ± 0.44 4.65 ± 0.50	0.09 0.11	0.042 0.024	0.001 0.000		
**1.2 Hygiene**	4.75 ± 0.45	0.09	0.135	0.086		
1.2.1 The living environment is dirty and disorderly	4.60 ± 0.50	0.11	0.068	0.002		
1.2.2 Poor ventilation **1.3 Housing** 1.3.1 No elevator 1.3.2 Insufficient sports living space 1.3.3 Lack of network/device	4.55 ± 0.51 3.95 ± 0.60 3.95 ± 0.69 3.80 ± 0.77 4.90 ± 0.31	0.11 0.15 0.17 0.20 0.06	0.068 0.041 0.007 0.004 0.030	0.002 0.010 0.000 0.000 0.000		
**2. Psychosocial field**	5.00 ± 0.00	0.00	0.250	-		
**2.1 Connection with community resources**	5.00 ± 0.00	0.00	0.098	0.025		
2.1.1 Resources are insufficient or cannot be obtained 2.1.2 Resource usage is Limited	4.60 ± 0.50 4.80 ± 0.41	0.11 0.09	0.019 0.030	0.000 0.001		
2.1.3 Unfamiliar with remote extended nursing acquisition procedures	4.80 ± 0.41	0.09	0.049	0.001		
**2.2 Mental health**	4.65 ± 0.49	0.11	0.040	0.010		
2.2.1 Sadness/depression	4.70 ± 0.47	0.10	0.010	0.000		
2.2.2 Fear/anxiety	4.70 ± 0.47	0.10	0.010	0.000		
2.2.3 Loneliness/fatigue	4.70 ± 0.47	0.10	0.010	0.000		
2.2.4 Irritability and irritability	4.70 ± 0.47	0.10	0.010	0.000		
**2.3 Family care/emotional support**	5.00 ± 0.00	0.00	0.098	0.025		
2.3.1 The patient’s physical care/safety is not guaranteed	4.60 ± 0.50	0.11	0.164	0.004		
2.3.2 Lack of emotional support/nurturing 2.3.3 Lack of proper symptom monitoring 2.3.4 Lack of medication and diet management 2.3.5 Lack of professional knowledge of caregivers 2.3.6 The caregiver’s caring burden is too heavy **2.4 Interpersonal relationship** 2.4.1 Receiving visits from friends and relatives 2.4.2 Participate in social activities normally 2.4.3 Communication difficulties with family, friends and colleagues 2.4.4 Show sensitivity to strangers	4.60 ± 0.50 4.60 ± 0.50 4.60 ± 0.50 4.60 ± 0.50 4.60 ± 0.50 3.90 ± 0.64 4.00 ± 0.56 3.95 ± 0.51 3.95 ± 0.51 3.95 ± 0.51	0.11 0.11 0.11 0.11 0.11 0.16 0.16 0.13 0.13 0.13	0.164 0.164 0.164 0.164 0.164 0.014 0.004 0.004 0.004 0.004	0.004 0.004 0.004 0.004 0.004 0.004 0.000 0.000 0.000 0.000		
**3 Physiological fields**	5.00 ± 0.00	0.00	0.25	-		
**3.1 Cycle**	4.65 ± 0.49	0.11	0.033	0.008		
3.1.1 Peripheral edema	4.40 ± 0.50	0.11	0.005	0.000		
3.1.2 Abnormal blood pressure	4.60 ± 0.50	0.11	0.008	0.000		
3.1.3 Dyslipidemia 3.1.4 Abnormal blood sugar	4.40 ± 0.50 4.80 ± 0.41	0.11 0.09	0.005 0.016	0.000 0.000		
**3.2 Digestion - hydration**	4.50 ± 0.51	0.11	0.017	0.004		
3.2.1 Nausea/vomiting/bloating	4.40 ± 0.50	0.13	0.007	0.000		
3.2.2 Disorder of laboratory indicators 3.2.3 Abnormal appetite 3.2.4 Balance of input and output **3.3 Excretion function** 3.3.1 Diarrhea 3.3.2 Constipation 3.3.3 Frequent/urgent urination 3.3.4 Abnormal urine volume	4.40 ± 0.50 4.40 ± 0.50 4.40 ± 0.50 4.60 ± 0.50 4.90 ± 0.31 4.40 ± 0.50 4.30 ± 0.47 4.30 ± 0.47	0.13 0.11 0.11 0.11 0.06 0.11 0.11 0.11	0.003 0.003 0.003 0.025 0.013 0.005 0.003 0.003	0.000 0.000 0.000 0.006 0.000 0.000 0.000 0.000		
**3.4 Skin numbness**	4.85 ± 0.37	0.07	0.045	0.011		
3.4.1 Skin numbness in other parts	4.20 ± 0.70	0.17	0.008	0.000		
3.4.2 Skin scar numbness at the surgical incision	5.00 ± 0.00	0.00	0.038	0.003		
**3.5 Infection** 3.5.1 Fungal infection 3.5.2 Bacterial infection 3.5.3 Other Infections **3.6 Oral hygiene** 3.6.1 Oral mucosal integrity 3.6.2 Tooth decay 3.6.3 Oral ulcers 3.6.4 Pain/swelling/bleeding gums 3.6.5 Gingivitis/Periodontitis **4 Health behavior related fields** **4.1 Nutrition** 4.1.1 Daily body requirement 4.1.2 Daily body intake 4.1.3 BMI index **4.2 Sleep and rest patterns** 4.2.1 Difficulty falling asleep 4.2.2 Sitting up at night 4.2.3 Insufficient sleep and rest 4.2.4 Insomnia **4.3 Physical activity** 4.3.1 General physical activity without discomfort 4.3.2 Feeling chest tightness and fatigue after general physical activity 4.3.3 Discomfort such as chest tightness after mild activity 4.3.4 Discomfort symptoms such as chest tightness still occur during rest 4.3.5 Lack of exercise program **4.4 Knowledge** 4.4.1 Describe postoperative dietary precautions 4.4.2 Describe the precautions for postoperative rehabilitation exercise 4.4.3 Describe the precautions for postoperative condition monitoring 4.4.4 Describe the precautions for postoperative drug management **4.5 Substance abuse** 4.5.1 Alcoholism 4.5.2 Smoking 4.5.3 Other bad eating habits **4.6 Drug treatment compliance** 4.6.1 Failure to take medication as prescribed 4.6.2 Drug side effects occur 4.6.3 Lack of drug expertise 4.6.4 Improper storage of drugs **4.7 Health status supervision** 4.7.1 Not receiving extended care 4.7.2 Failure to return for examination as required	4.80 ± 0.41 4.95 ± 0.22 5.00 ± 0.00 5.00 ± 0.00 4.85 ± 0.37 5.00 ± 0.00 4.60 ± 0.50 4.95 ± 0.22 4.60 ± 0.50 4.60 ± 0.50 5.00 ± 0.00 4.85 ± 0.37 4.60 ± 0.50 5.00 ± 0.00 5.00 ± 0.00 4.45 ± 0.60 4.85 ± 0.37 4.20 ± 0.41 4.85 ± 0.37 4.20 ± 0.41 4.40 ± 0.50 4.55 ± 0.51 4.25 ± 0.44 4.25 ± 0.44 4.25 ± 0.44 4.35 ± 0.59 4.00 ± 0.56 4.30 ± 0.66 4.30 ± 0.66 4.30 ± 0.66 4.40 ± 0.60 4.85 ± 0.37 4.85 ± 0.37 4.55 ± 0.51 4.90 ± 0.31 4.95 ± 0.22 5.00 ± 0.00 4.95 ± 0.22 4.80 ± 0.41 4.40 ± 0.50 4.25 ± 0.44 4.35 ± 0.49 4.35 ± 0.49	0.08 0.04 0.00 0.00 0.08 0.00 0.11 0.04 0.11 0.11 0.00 0.08 0.11 0.00 0.00 0.13 0.08 0.10 0.08 0.10 0.11 0.11 0.10 0.10 0.10 0.14 0.14 0.15 0.15 0.15 0.14 0.08 0.08 0.11 0.06 0.04 0.00 0.04 0.09 0.11 0.10 0.11 0.11	0.063 0.021 0.021 0.021 0.067 0.026 0.007 0.019 0.007 0.007 0.250 0.042 0.006 0.018 0.018 0.025 0.010 0.003 0.010 0.003 0.020 0.007 0.002 0.002 0.004 0.005 0.009 0.002 0.002 0.002 0.002 0.057 0.031 0.008 0.020 0.080 0.027 0.027 0.016 0.007 0.016 0.008 0.008	0.016 0.000 0.000 0.000 0.017 0.000 0.000 0.000 0.000 0.000 - 0.011 0.000 0.000 0.000 0.006 0.000 0.000 0.000 0.000 0.005 0.000 0.000 0.000 0.000 0.000 0.002 0.000 0.000 0.000 0.000 0.014 0.000 0.000 0.000 0.020 0.001 0.001 0.000 0.000 0.004 0.000 0.000		

## Discussion

### Scientific and reliability analysis of index construction

Through literature review and clinical research, this study use of Omaha question classification system, through the enquiry for two rounds of the Delphi method, build the index system of nursing sensitivity in elderly patients with liver transplantation outcome. This study provides a solid theoretical basis for extending nursing services to patients and has a strong scientific basis. The effective recovery rate of the two rounds of questionnaires was 100%, and the experts in each round could put forward constructive opinions, indicating that the experts had good participation and attention. The authority coefficients of the two rounds of consultation were 0.904 and 0.905, respectively, indicating that the experts were familiar with the content of the field and the judgment basis was strong. The Kendall coordination coefficients of the first-level, second-level and third-level indicators obtained by expert consultation were 0.419, 0.418 and 0.394, respectively. The mean of each index was ≥ 3.50, and the coefficient of variation was < 0.25, which indicated that the expert opinions tended to be unified and the reliability was good.

### Index construction can be comprehensive and targeted

#### Environment field

Environment field is the most neglected but extremely important. Zhao QF et al. [12] point out that income and hygiene are the key factors affecting the prognosis and quality of life of elderly patients. The same argument was made in our study, the income and hygiene also ranks the top two in the environment field. “Internet + Extended nursing service” can enable medical staff to guide elderly patients’ postoperative self-management remotely and effectively [13]. “Internet + Extended nursing service” can improve the ideological consciousness of elderly patients through popular science education and other forms, and fundamentally eliminate unclean practices and dirty environments. However, at present in China, many remote and rural areas [14] still lack network signals and terminal equipment. Health education resources for elderly patients after transplantation are very limited, and the source of income is also uncertain. Therefore, the national government also needs to improve medical insurance [15], remote service [16] and other aspects, so as to reduce the economic pressure of elderly patients. To improve health conditions, the weight value of the third-level indicator “lack of network/device " in this study is 0.030, which is much higher important than other indicators, which also indicates that experts believe that the construction of telemedicine services is very important, which is also in line with the development trend of “Internet + Extended nursing services”.

#### Psychological field

In the psychology field, the average scores of “connection with community resources” and “family care/emotional support” both reached 5.00 ± 0.00, ranking the top two in this field, indicating that experts attach great importance to these two indicators. At present, the construction of advanced nursing practice model of liver transplantation is still being explored in China. The community medical service resources available to postoperative patients are very limited [17], resulting in heavy burden of family care, especially for elderly patients due to lack of knowledge, low self-management behavior and other reasons, which aggravate the burden of family care. In contrast to developed countries, they have formed a hospital-community-family integrated nursing pattern. The problem of home care for liver transplant patients can be systematically solved [18].

The weight of the outcome indicator “mental health” was 0.040, ranking the third. Gu Yanmei et al. have shown [19] that the mental health of elderly liver transplant patients is at a low level. Moreover, the degree of mental health is closely related to family care [20] and social support [21]. State-owned literature also pointed out that the mental health problems of elderly liver transplant patients after surgery may be closely related to the lack of community medical resources [22] and family care [23]. Therefore, in order to truly solve the social and psychological problems of patients, it is necessary for the state to increase the construction of primary medical units and build the construction of hospital-community-family medical union, to meet the social and psychological needs of elderly liver transplant patients.

#### Physiological field

In the physiology field, the three most prominent indicators are “oral hygiene,” “infection,” and “skin numbness,” with respective values of 0.067, 0.063, and 0.045. Notably, “oral hygiene” holds the highest weight ratio among them It is well known that the oral cavity serves as a primary site for bacterial colonization. Prolonged usage of immunosuppressive drugs like rapamycin may result in oral ulcers and compromise the integrity of oral mucosa [24]. Only by maintaining proper oral hygiene can infections be prevented effectively. Furthermore, the weight values of the three-level indexes “oral ulcer” and “oral mucosal integrity” also ranked first and second, indicating that the experts’ views were consistent with those of the literature.

The reason for the analysis of “infection” index may be that the T lymphocyte immune response of liver transplant patients after long-term immunosuppressants is suppressed, and the immune function of elderly patients is lower than that of the general population. Moreover, under the influence of the novel coronavirus epidemic for three years, elderly patients are more likely to be infected, which is also consistent with the research results of Cui Heng et al. [25].

“Skin numbness” may be related to peripheral nerve injury and ischemia in the operative area [26]. The weight and combined weight of the subordinate tertiary index “skin scar numbness at surgical incision” rank first in this field, which also indicates that experts attach great importance to the problem of postoperative local skin numbness and unconsciousness of patients.

The “abnormal blood sugar " and “diarrhea” are indicators with high weight value in this field, indicating that experts attach great importance to these two indicators. Yujian Z et al. [27] also point out that hyperglycemia and diarrhea are also the most common complications of elderly liver transplant patients at home after surgery. Therefore, the government of China need to increase the intelligent construction of primary medical units, so that medical staff can use the network to achieve linkage management. In order to better self-monitoring of elderly liver transplant patients at home.

#### Health behavior related fields

The only health behavior field with an average score of 5.00 ± 0.00 was “drug treatment compliance”, highlighting the significant emphasis experts place on postoperative medication for elderly liver transplant patients. It also indicates that poor drug compliance is relatively common in the substantive organ transplantation field [28]. Domestic studies [29] show that 39.40-78.38% of liver transplant recipients have poor medication compliance, which may be attributed to elderly liver transplant patients’ old age, memory loss, and the variety, quantity and duration of medication taken at home after surgery. In the absence of supervision by medical staff, there is a sense of burnout, and occasionally in the case of missing medication, there is no significant change in physical function. In the long run, the patient will let his guard down in his mind, leading to low adherence to medication.

In addition, in this field, the average scores of “nutrition” and “substance abuse” are also greater than 4.50 ± 0.00, indicating that experts attach great importance to these issues, which is also consistent with Fuchi Yang et al. [30]. It indicate that patients’ overall self-management behavior at home needs to be improved. It is more necessary for the state to increase the intelligent construction of grass-roots medical units, so that medical staff can use the network to achieve linkage management, and build the construction of hospital-community-family medical union, so as to meet the needs of patients’ health behaviors.

### Indicators have clinical applicability

When implementing the evaluation of the sensitivity outcome index system of home care for elderly liver transplant patients established in this study, medical staff should also receive standardized training and convert the indicators into plain language to ask patients, so as to integrate the core nursing problems faced by patients at home. In addition, the standardized expression of nursing problems in our study was mostly adopted in the Omaha problem classification system, which laid the foundation for the establishment of a unified language for electronic nursing information system, the unification of the expression of nursing information between different institutions and different staff, and the creation of high-quality “Internet + nursing” service.

## Conclusion

Based on the Omaha problem classification combined with Delphi method, our study constructed a sensitivity outcome index system for home nursing of elderly liver transplant patients, providing a theoretical basis for nursing staff to build an accurate individualized continued nursing model. However, due to the lack of knowledge and time of researchers, the effectiveness and effectiveness of indicators need to be continuously adjusted in clinical application, and the combination of Internet technology in extended care to further improve patients’ self-management ability is also a future development trend.

### Electronic supplementary material

Below is the link to the electronic supplementary material.

Supplementary Material 1

## Data Availability

The datasets used or analysed during the current study are available from the corresponding author on reasonable request.

## References

[1] Yu Ying. Construction of standardized nursing system for organ donor liver transplantation [J]. Chin Nurs Manage 2018,18(S1):60–2.

[2] Zhou Xia translated, Zhang Min proofread. Evaluation for liver transplantation in adults:2013 Practice Guideline by the American Association for the Study of Liver Diseases and the American Society of Transplantation[J]. J Clin Hepatobiliary Dis. 2014;000(006):583–5.

[3] Xue Zhao T, Min D, Yan-Ying, et al. Application of remote follow-up management system in extended care of liver transplant recipients [J]. J Nurs Sci. 2019;34(21):73–5.

[4] Caixia Z, Zhixian F. Construction of perioperative nursing sensitivity index for liver transplantation [J]. Chin J Practical Nurs. 2018;34(27):2120–6.

[5] Rui Y, Xiu-Xin M, Han-Wen C. Construction of nursing problem evaluation system for elderly patients with Type 2 diabetes mellitus [J]. Journal of Nursing, 2007.,2017,24(4): 6–10.

[6] Zhang Mei X, Na ZHU, Aifang. Evaluation of care for patients with inflammatory bowel disease based on Omaha Problem classification system [J]. Qilu Nurs J. 2019;25(22):9–12.

[7] Yingxia L, Xiaojia ZHU, Lingxiao WANG. Evaluation index of nursing outcome after radical resection of bladder cancer in elderly patients [J]. J Nurs Adm. 2020;20(5):345–9.

[8] Tomotaki A, Iwamoto T, Yokota S, Research Types and New Trends on the Omaha System Published. From 2012 to 2019: a scoping Review[J]. Volume 40. CIN-COMPUT INFORM NU; 2022. pp. 531–7. 8. 10.1097/CIN.0000000000000887 35929744

[9] Walling AM, Ahluwalia SC, Wenger NS. Palliative Care Quality indicators for patients with end-stage liver Disease due to Cirrhosis[J].DIGEST DIS SCI,2017,62(1):84–92. 10.1007/s10620-016-4339-3 PMC5384571 27804005

[10] Sun Huihui ZHANG, Bingliang L. Construction of pre-operation rehabilitation program for liver transplantation patients [J]. Chin J Nurs. 2023;58(18):2195–202.

[11] Tong S, Lili WEI, Guofang KUANG. Construction and verification of health literacy evaluation index system of gestational diabetes mellitus patients [J]. Chin J Nurs. 2023;58(5):565–71.

[12] Zhao QF, Wang J, Nicholas S, et al. Health-related quality of life and health service use among multimorbid middle-aged and older-aged adults in China:a cross-sectional study in Shandong Province[J]. Int J Environ Res Public Health. 2020;17(24):E9261. 10.3390/ijerph17249261 PMC7764479 33322307

[13] Xu Ling C. Construction and implementation of internet + nursing service model with hospital as the main body [J]. J Nurs Sci. 2020;35(11):1–5.

[14] Tian Yutong Z, Xiaohua YH. Research on construction and application of internet + nursing service platform [J]. Chin J Nurs 2020,55(10):1537–42.

[15] Osenenko KM, Kuti E, Deighton AM, et al. Burden of hospitalization for heart failure in the United States:a systematic literature review[J]. J Manag Care Spec Pharm. 2022;28(2):157–67. 35098748 10.18553/jmcp.2022.28.2.157 PMC10373049

[16] Shujing H, Yi WANG, Bao GAO. Review on the scope of remote intervention in the continuous care of lung transplant patients [J]. Chin J Nurs. 2023;58(14):1773–9.

[17] Feng Zhixian SHEN, Mingyan LU, Jianfang. Construction and implementation effect of advanced nursing practice model for liver transplantation [J]. Chin Nurs Manage. 2023;23(5):650–3.

[18] Chen Xiayu ZHOU, Jingfen HUA, Haiying. The Development status of continuous nursing model in the United States and its implications for China [J]. Nurs Res. 2021;35(18):3293–7.

[19] Gu Yanmei Z, Lili X, Shuangmei. Application of AIDET communication model combined with psychological intervention in patients after liver transplantation [J]. Nursing research,2022,37(12):2286–8.

[20] Guo Zhongxian GOU, Yuli SHA, Liyan. Effect of transitional nursing program on migration stress of family members of liver transplant patients [J]. Chin J Acute Crit Care. 2023;4(2):101–5.

[21] Guo Limin LI, Lezhi LU, Yanfang. The mediating effect of self-efficacy on social support and psychological resilience of liver transplant recipients [J]. Chin Nurs Educ. 2021;18(2):173–7.

[22] Auerbach AD, Wachter RM, Katz P et al. Implementation of a Voluntary Hospitalist Service at a community Teaching Hospital[J].Annals of internal medicine, 2003, 137(11):859–65.10.7326/0003-4819-137-11-200212030-00006. 10.7326/0003-4819-137-11-200212030-00006 12458985

[23] Bolden L, Wicks MN. The clinical utility of the stress process model in family caregivers of liver transplant candidates[. J] Progress Transplantation. 2008;18(2):74. 10.7182/prtr.18.2.d1737751t465u63q. 10.7182/prtr.18.2.d1737751t465u63q 18615971

[24] Cristiana A, D,RalucaPaula V,Corien P et al. Oral diseases after liver transplantation: a systematic review and meta-analysis.[J]. British dental journal,2021,231(2). 10.1038/s41415-021-3219-1 34302095

[25] Cui Heng W, Xuerui F, Qiaomei, et al. Home nursing of liver transplant patients during the Novel coronavirus pneumonia epidemic [J]. Nurs Res. 2020;34(05):741–3.

[26] Zhang B. Construction of nursing quality evaluation index system and preliminary application of index monitoring after adult liver transplantation [D]. Qingdao University,2022.10.27262/d.cnki.gqdau.2022.000105.

[27] Yujian Z, Qing C, Lishan P et al. Related Factors of Hepatocellular Carcinoma Recurrence Associated With Hyperglycemia After Liver Transplantation[J]. Transplantation Proceedings,2020,53(1). 10.1016/j.transproceed.2020.10.027 33272654

[28] Yabin S, Hongxia L, Lu W et al. Immunosuppressive medication adherence in liver transplant recipients[J]. Chin Nurs Manage, 2017.

[29] Gong Yueqiao L, Guofang RAO, Wei et al. Effect of mind mapping on medication compliance of liver transplant recipients [J]. Journal of Nursing Care, 2007. 2020,27(22):1–3.10.16460/j.issn1008-9969.2020.22.001.

[30] Yang FC, Chen HM, Huang CM et al. The difficulties and needs of Organ Transplant recipients during postoperative care at home: a systematic Review[J]. Int J Environ Res Public Health, 17(16)2023-10.3390/ijerph17165798. 10.3390/ijerph17165798 PMC7459921 32796529

