# Supplementary material for: The sensitivity outcome index system for home care of elderly liver transplant patients was developed based on the Omaha problem classification system
Source: BMC Med Inform Decis Mak. 2024 Jul 25;24:207. doi: 10.1186/s12911-024-02617-w (PMC11270964; doi:10.1186/s12911-024-02617-w)
Supplement: Supplementary file 1 — Supplementary Material 1 [file 12911_2024_2617_MOESM1_ESM.docx]

Based on the Omaha question classification system, an expert consultation questionnaire on nursing sensitivity outcome index system for elderly liver transplantation patients was constructed

Dear Expert:

Thank you very much for taking time out of your busy schedule to review this expert consultation questionnaire!

Based on the characteristics of elderly liver transplant patients and the Omaha problem classification system, this study carried out "Construction of nursing sensitivity outcome index system for elderly liver transplant patients based on the Omaha problem classification system". In view of your high academic attainments in this field, we sincerely invite you to be the consulting expert of this research. Please give your opinions or suggestions in your busy time, which is very important for our research. This study will consult experts through email questionnaires, thank you for your support and help!

This consultation is scheduled for two rounds initially. Due to the needs of the project, please send back to the email: 893458905@qq.com within one week. If you have any questions about the content of this questionnaire, please feel free to contact us.

Researcher: Bin Wang

Contact: 17853299575

Part I: Questionnaire of basic situation of experts

Note: Please fill in the corresponding box according to your actual situation.

| Name |  | Gender |  |
| --- | --- | --- | --- |
| Age |  | Highest degree |  |
| Technical title |  | Working years |  |
| Professional field |  | | |
| Supervisor or not | □yes □no | □Master's supervisor □Doctor’s supervisor | |
| Types of supervising graduate students | □Academic □Professional □Both | | |
| Contact information | E-mail： Phone: | | |

Part two: Expert familiarity and judgment basis questionnaire

1. The expert's familiarity with the consultation content

Note: Please mark "√" in the corresponding space according to your familiarity with the following fields of expertise.

| Content category | Familiarity | | | | |
| --- | --- | --- | --- | --- | --- |
|  | Very（1） | More  （0.8） | Average（0.6） | Poor  （0.4） | Low  （0.2） |
| Aged care |  |  |  |  |  |
| Omaha system |  |  |  |  |  |
| Nursing sensitivity index |  |  |  |  |  |
| Liver transplantation |  |  |  |  |  |

2. The basis and degree of expert's judgment on the content of consultation

Note: According to the degree of influence of each basis on your judgment, please mark "√" in the corresponding space.

| Judgment basis | Large | Medium | Small |
| --- | --- | --- | --- |
| Theoretical analysis |  |  |  |
| Practical experience |  |  |  |
| Refer to guides and literature |  |  |  |
| Intuitive feeling |  |  |  |

Part III: Based on the Omaha question classification system, the first consultation draft of the nursing sensitivity outcome index system for elderly liver transplantation patients was constructed

Each item is divided into five levels according to its importance, with 1-5 points representing the least to the most important: please click the space to mark √ on the corresponding value according to your own judgment (only select one answer for each item). If you have other suggestions for this part of the content, you can write the suggestion of revision to the rear comments.

Table 1: To construct the first round of first-level item evaluation table of nursing sensitivity outcome index system for elderly liver transplant patients based on the Omaha question classification system, please tick √ in the expert opinion column, and indicate in the corresponding space if you have any modification suggestions or additional items.

| First-level entry | Expert opinion | | | | | Modification suggestion |
| --- | --- | --- | --- | --- | --- | --- |
|  | Great importance  5 | Importance 4 | General 3 | Insignificance  2 | Very unimportant  1 |  |
| 1. Environmental field |  |  |  |  |  |  |
| 2. Psychosocial field |  |  |  |  |  |  |
| 3. Physical areas |  |  |  |  |  |  |
| 4. Health behavior-related fields |  |  |  |  |  |  |

Table 2: The first round of second-level item evaluation table of nursing sensitivity outcome index system for elderly liver transplant patients is constructed based on Omaha problem classification system. Please tick √ in the expert opinion column. If you have any modification comments or additional items, please note in the corresponding space

| First-level entry | Second-level entry | Expert opinion | | | | | Modification suggestion |
| --- | --- | --- | --- | --- | --- | --- | --- |
|  |  | Great importance  5 | Importance 4 | General 3 | Insignificance  2 | Very unimportant  1 |  |
| 1. Environmental field | 1.1 Income |  |  |  |  |  |  |
|  | 1.2 Hygiene |  |  |  |  |  |  |
|  | 1.3 Dwelling |  |  |  |  |  |  |
| 2. Social psychological field | 2.1 Links to community resources |  |  |  |  |  |  |
|  | 2.2 Mental health |  |  |  |  |  |  |
|  | 2.3 Home care/emotional support |  |  |  |  |  |  |
|  | 2.4 Grieved |  |  |  |  |  |  |
|  | 2.5 Interpersonal relationship |  |  |  |  |  |  |
| 3. Physiological field | 3.1 Loop |  | √ |  |  |  |  |
|  | 3.2 Digestion - hydration |  |  |  |  |  |  |
|  | 3.3 Excretory function |  |  |  |  |  |  |
|  | 3.4 Infection |  |  |  |  |  |  |
|  | 3.5 Oral hygiene |  |  |  |  |  |  |
| 4. Health behavior related fields | 4.1 Nourishment |  |  |  |  |  |  |
|  | 4.2 Sleep and rest patterns |  |  |  |  |  |  |
|  | 4.3 Physical activity |  |  |  |  |  |  |
|  | 4.4 Substance abuse |  |  |  |  |  |  |
|  | 4.5 Adherence to medication |  |  |  |  |  |  |
|  | 4.6 Health supervision |  |  |  |  |  |  |
|  | 4.7 Care/childcare |  |  |  |  |  |  |

Table 3: The first round of three-level item evaluation table of nursing sensitivity outcome index system for elderly liver transplant patients is constructed based on the Omaha question classification system. Please tick √ in the expert opinion column. If you have any modification suggestions or additional items, please indicate in the corresponding space.

| Articles by level entry | | | Degree of importance | | | | | Modification suggestion |
| --- | --- | --- | --- | --- | --- | --- | --- | --- |
| First-level entry | Second-level entry | Three-level entry | Great importance | Importance | General | Insignificance | Very unimportant |  |
|  |  |  | 5 | 4 | 3 | 2 | 1 |  |
| 1. Environmental field | 1.1 Income | 1.1.1 Low/no income |  |  |  |  |  |  |
|  |  | 1.1.2 No health insurance. |  |  |  |  |  |  |
|  |  | 1.1.3 Income does not cover medical expenses |  |  |  |  |  |  |
|  | 1.2 Hygiene | 1.2.1 The living environment is dirty and bad |  |  |  |  |  |  |
|  |  | 1.2.2 Poor ventilation |  |  |  |  |  |  |
|  | 1.3 Dwelling | 1.3.1 No lift |  |  |  |  |  |  |
|  |  | 1.3.2 Insufficient sports living space |  |  |  |  |  |  |
| 2. Social psychological field | 2.1 Links to community resources | 2.1.1 Insufficient resources/unavailable resources  2.1.2 Limited use resources |  |  |  |  |  |  |
|  |  | 2.1.3 Unfamiliar with remote extension care access procedures |  |  |  |  |  |  |
|  | 2.2 Mental health | 2.2.1 Sadness/depression |  |  |  |  |  |  |
|  |  | 2.2.2 Fear/anxiety |  |  |  |  |  |  |
|  |  | 2.2.3 Apathy/fatigue |  |  |  |  |  |  |
|  |  | 2.2.4 Irascibility |  |  |  |  |  |  |
|  | 2.3 Home care/emotional support | 2.3.1 Lack of patient physical care/safety |  |  |  |  |  |  |
|  |  | 2.3.2 Lack of emotional support/nurturing |  |  |  |  |  |  |
|  |  | 2.3.3 Lack of proper symptom monitoring |  |  |  |  |  |  |
|  |  | 2.3.4 Lack of medication, diet management |  |  |  |  |  |  |
|  |  | 2.3.5 Insufficient professional knowledge of caregivers |  |  |  |  |  |  |
|  |  | 2.3.6 The caregiver has too much care burden |  |  |  |  |  |  |
|  | 2.4 Grieved | 2.4.1 Denial of fact |  |  |  |  |  |  |
|  |  | 2.4.2 Anger/self-harming tendencies |  |  |  |  |  |  |
|  |  | 2.4.3 Bargain |  |  |  |  |  |  |
|  |  | 2.4.4 Depression/depression |  |  |  |  |  |  |
|  |  | 2.4.5 Accept |  |  |  |  |  |  |
|  | 2.5 Interpersonal relationship | 2.5.1 Receive visits from friends and relatives |  |  |  |  |  |  |
|  |  | 2.5.2 Participate in social activities as normal |  |  |  |  |  |  |
|  |  | 2.5.3 Difficulty communicating with family, friends and colleagues |  |  |  |  |  |  |
|  |  | 2.5.4 Show sensitivity to strangers |  |  |  |  |  |  |
| 3. Physiological field | 3.1 Loop | 3.1.1 Edema |  |  |  |  |  |  |
|  |  | 3.1.2 Abnormal blood pressure |  |  |  |  |  |  |
|  |  | 3.1.3 Dyslipemia |  |  |  |  |  |  |
|  |  | 3.1.4 Dysglycemia |  |  |  |  |  |  |
|  | 3.2 Digestion - hydration | 3.2.1 Indigestion |  |  |  |  |  |  |
|  |  | 3.2.2 Electrolyte disturbance |  |  |  |  |  |  |
|  |  | 3.2.3 Anorexia |  |  |  |  |  |  |
|  |  | 3.2.4 Bad habits/vomiting/bloating |  |  |  |  |  |  |
|  | 3.3 Excretory function | 3.3.1 Diarrhea |  |  |  |  |  |  |
|  |  | 3.3.2 Constipation |  |  |  |  |  |  |
|  |  | 3.3.3 Frequent and urgent urination |  |  |  |  |  |  |
|  |  | 3.3.4 Abnormal urine volume |  |  |  |  |  |  |
|  | 3.4 Skin numbness | 3.4.1 Other parts of the skin numb |  |  |  |  |  |  |
|  |  | 3.4.2 The skin scar at the surgical incision is numb |  |  |  |  |  |  |
|  | 3.5 Infection | 3.5.1 Fungal infection |  |  |  |  |  |  |
|  |  | 3.5.2 Bacterial infection |  |  |  |  |  |  |
|  |  | 3.5.3 Other infections |  |  |  |  |  |  |
|  | 3.6 Oral hygiene | 3.6.1 Oral mucosal integrity |  |  |  |  |  |  |
|  |  | 3.6.2 Decayed tooth |  |  |  |  |  |  |
|  |  | 3.6.3 Oral ulcer |  |  |  |  |  |  |
|  |  | 3.6.4 Pain/swelling/bleeding gums |  |  |  |  |  |  |
|  |  | 3.6.5牙龈炎/牙周炎 |  |  |  |  |  |  |
| 4. Health behavior related fields | 4.1 Nourishment | 4.1.1 Daily body requirement |  |  |  |  |  |  |
|  |  | 4.1.2 Daily body intake |  |  |  |  |  |  |
|  |  | 4.1.3 BMI index |  |  |  |  |  |  |
|  | 4.2 Sleep and rest patterns | 4.2.1 Difficulty falling asleep |  |  |  |  |  |  |
|  |  | 4.2.2 Sit up at night |  |  |  |  |  |  |
|  |  | 4.2.3 Lack of sleep rest |  |  |  |  |  |  |
|  |  | 4.2.4 Insomnia |  |  |  |  |  |  |
|  | 4.3 Physical activity | 4.3.1 General physical activity without discomfort |  |  |  |  |  |  |
|  |  | 4.3.2 General physical activity after feeling chest tightness, fatigue |  |  |  |  |  |  |
|  |  | 4.3.3 Discomfort such as chest tightness after mild activity |  |  |  |  |  |  |
|  |  | 4.3.4 Chest tightness and other uncomfortable symptoms still exist at rest |  |  |  |  |  |  |
|  |  | 4.3.5 Lack of exercise program |  |  |  |  |  |  |
|  | 4.4 Care/childcare | 4.4.1 Inability to self-care |  |  |  |  |  |  |
|  |  | 4.4.2 An unwillingness to take care of yourself |  |  |  |  |  |  |
|  |  | 4.4.3 Can't care for your family |  |  |  |  |  |  |
|  |  | 4.4.4 Unwilling to care for family members |  |  |  |  |  |  |
|  | 4.5 Substance abuse | 4.5.1 Intemperance |  |  |  |  |  |  |
|  |  | 4.5.2 Smoking |  |  |  |  |  |  |
|  |  | 4.5.3 Other bad eating habits |  |  |  |  |  |  |
|  | 4.6 Other bad eating habits | 4.6.1 Not taking medication as prescribed |  |  |  |  |  |  |
|  |  | 4.6.2 Have drug side effects |  |  |  |  |  |  |
|  |  | 4.6.3 Lack of drug expertise |  |  |  |  |  |  |
|  |  | 4.6.4 Improper drug storage |  |  |  |  |  |  |
|  | 4.7 Health supervision | 4.7.1 Did not receive extended medical care |  |  |  |  |  |  |
|  |  | 4.7.2 Did not follow up as requested |  |  |  |  |  |  |
|  |  | 4.7.3 Failed to follow up as requested |  |  |  |  |  |  |

Based on the Omaha question classification system, an expert consultation questionnaire on nursing sensitivity outcome index system for elderly liver transplantation patients was constructed

Dear expert,

Thank you very much for taking time out of your busy schedule to review this expert consultation questionnaire!

Based on the characteristics of elderly liver transplant patients and the Omaha problem classification system, this study carried out "Construction of nursing sensitivity outcome index system for elderly liver transplant patients based on the Omaha problem classification system". In view of your high academic attainments in this field, we sincerely invite you to be the consulting expert of this research. Please give your opinions or suggestions in your busy time, which is very important for our research. This study will consult experts through email questionnaires, thank you for your support and help!

This consultation is scheduled for two rounds initially. Due to the needs of the project, please send back to the email: 893458905@qq.com within one week. If you have any questions about the content of this questionnaire, please feel free to contact us.

Researcher: Bin Wang

Contact: 17853299575

Part I: Questionnaire of basic situation of experts

Note: Please fill in the corresponding box according to your actual situation.

| Name |  | Gender |  |
| --- | --- | --- | --- |
| Age |  | Highest degree |  |
| Technical title |  | Working years |  |
| Professional field |  | | |
| Supervisor or not | □yes □no | □Master's supervisor □Doctor’s supervisor | |
| Types of supervising graduate students | □Academic □Professional □Both | | |
| Contact information | E-mail： Phone: | | |

Part two: Expert familiarity and judgment basis questionnaire

1. The expert's familiarity with the consultation content

Note: Please mark "√" in the corresponding space according to your familiarity with the following fields of expertise.

| Content category | Familiarity | | | | |
| --- | --- | --- | --- | --- | --- |
|  | Very（1） | More  （0.8） | Average（0.6） | Poor  （0.4） | Low  （0.2） |
| Aged care |  |  |  |  |  |
| Omaha system |  |  |  |  |  |
| Nursing sensitivity index |  |  |  |  |  |
| Liver transplantation |  |  |  |  |  |

2. The basis and degree of expert's judgment on the content of consultation

Note: According to the degree of influence of each basis on your judgment, please mark "√" in the corresponding space.

| Judgment basis | Large | Medium | Small |
| --- | --- | --- | --- |
| Theoretical analysis |  |  |  |
| Practical experience |  |  |  |
| Refer to guides and literature |  |  |  |
| Intuitive feeling |  |  |  |

Part III: Based on the Omaha question classification system, the second consultation draft of the nursing sensitivity outcome index system for elderly liver transplantation patients was constructed

Each item is divided into five levels according to its importance, with 1-5 points representing the least to the most important: please click the space to mark √ on the corresponding value according to your own judgment (only select one answer for each item). If you have other suggestions for this part of the content, you can write the suggestion of revision to the rear comments.

Table 1: The second round of first-level item evaluation table of nursing sensitivity outcome index system for elderly liver transplant patients is constructed based on the Omaha question classification system. Please tick √ in the expert opinion column. If you have any modification or addition, please indicate it in the corresponding space.

| First-level entry | Expert opinion | | | | | Modification suggestion |
| --- | --- | --- | --- | --- | --- | --- |
|  | Great importance  5 | Importance 4 | General 3 | Insignificance  2 | Very unimportant  1 |  |
| 1. Environmental field |  |  |  |  |  |  |
| 2. Psychosocial field |  |  |  |  |  |  |
| 3. Physical areas |  |  |  |  |  |  |
| 4. Health behavior-related fields |  |  |  |  |  |  |

Table 2: The second round of second-level item evaluation table of nursing sensitivity outcome index system for elderly liver transplant patients is constructed based on the Omaha question classification system. Please tick √ in the expert opinion column. If you have any modification comments or additional items, please note in the corresponding space

| First-level entry | Second-level entry | Expert opinion | | | | | Modification suggestion |
| --- | --- | --- | --- | --- | --- | --- | --- |
|  |  | Great importance  5 | Importance 4 | General 3 | Insignificance  2 | Very unimportant  1 |  |
| 1. Environmental field | 1.1 Income |  |  |  |  |  |  |
|  | 1.2 Hygiene |  |  |  |  |  |  |
|  | 1.3 Dwelling |  |  |  |  |  |  |
| 2. Social psychological field | 2.1 Links to community resources |  |  |  |  |  |  |
|  | 2.2 Mental health |  |  |  |  |  |  |
|  | 2.3 Home care/emotional support |  |  |  |  |  |  |
|  | 2.4 Interpersonal relationship |  |  |  |  |  |  |
| 3. Physiological field | 3.1 Loop |  | √ |  |  |  |  |
|  | 3.2 Digestion - hydration |  |  |  |  |  |  |
|  | 3.3 Excretory function |  |  |  |  |  |  |
|  | 3.4 Sight and hearing |  |  |  |  |  |  |
|  | 3.5 Infect |  |  |  |  |  |  |
|  | 3.6 Oral hygiene |  |  |  |  |  |  |
| 4. Health behavior related fields | 4.1 Nutrition |  |  |  |  |  |  |
|  | 4.2 Sleep and rest patterns |  |  |  |  |  |  |
|  | 4.3 Physical Activity |  |  |  |  |  |  |
|  | 4.4 Knowledge |  |  |  |  |  |  |
|  | 4.5 Substance Abuse |  |  |  |  |  |  |
|  | 4.6 Drug treatment compliance |  |  |  |  |  |  |
|  | 4.7 Health Status Supervision |  |  |  |  |  |  |

Table 3: The second round of three-level item evaluation table of nursing sensitivity outcome index system for elderly liver transplant patients is constructed based on the Omaha question classification system. Please tick √ in the expert opinion column. If you have any modification or additional items, please indicate in the corresponding space.

| Articles by level entry | | | Degree of importance | | | | | | | | | | Modification suggestion | |
| --- | --- | --- | --- | --- | --- | --- | --- | --- | --- | --- | --- | --- | --- | --- |
| First-level entry | Second-level entry | Three-level entry | Great importance  5 | | Importance 4 | | | General 3 | Insignificance  2 | | Very unimportant  1 | |  | |
|  |  |  | 5 | | 4 | | | 3 | 2 | | 1 | |  |  |
| 1. Environmental field | 1.1 Income | 1.1.1 Low/no income |  | |  | | |  |  | |  | |  | |
|  |  | 1.1.2 No health insurance |  | |  | | |  |  | |  | |  |  |
|  |  | 1.1.3 Income does not cover medical expenses |  | |  | | |  |  | |  | |  |  |
|  | 1.2Hygiene | 1.2.1 The living environment is dirty and disorderly |  | |  | | |  |  | |  | |  | |
|  |  | 1.2.2 Poor ventilation |  | |  | | |  |  | |  | |  | |
|  | 1.3Housing | 1.3.1 No elevator |  | |  | | |  |  | |  | |  | |
|  |  | 1.3.2 Insufficient sports living space |  | |  | | |  |  | |  | |  | |
| 2. Social psychological field | 2.1 Links to community resources | 2.1.1 Resources are insufficient or cannot be obtained  2.1.2 Resource usage is Limited |  | |  | | |  |  | |  | |  | |
|  |  | 2.1.3 Unfamiliar with remote extended care acquisition procedures |  |  |  |  |  |  |  |  |  |  |  |  |
|  | 2.2 Mental health | 2.2.1 Sadness/depression |  |  |  |  |  |  |  |  |  |  |  |  |
|  |  | 2.2.2 Fear/anxiety |  |  |  |  |  |  |  |  |  |  |  |  |
|  |  | 2.2.3 Apathy/fatigue |  |  |  |  |  |  |  |  |  |  |  |  |
|  |  | 2.2.4 Irritability and irritability |  |  |  |  |  |  |  |  |  |  |  |  |
|  | 2.3 Home care/emotional support | 2.3.1 The patient's physical care/safety is not guaranteed |  |  |  |  |  |  |  |  |  |  |  |  |
|  |  | 2.3.2 Lack of emotional support/nurturing |  |  |  |  |  |  |  |  |  |  |  |  |
|  |  | 2.3.3 Lack of proper symptom monitoring |  |  |  |  |  |  |  |  |  |  |  |  |
|  |  | 2.3.4 Lack of medication and diet management |  |  |  |  |  |  |  |  |  |  |  |  |
|  |  | 2.3.5 Lack of professional knowledge of caregivers |  |  |  |  |  |  |  |  |  |  |  |  |
|  |  | 2.3.6 The caregiver's caring burden is too heavy |  |  |  |  |  |  |  |  |  |  |  |  |
|  | 2.4 Interpersonal relationship | 2.4.1 Receiving visits from friends and relatives |  |  |  |  |  |  |  |  |  |  |  |  |
|  |  | 2.4.2 Participate in social activities normally |  |  |  |  |  |  |  |  |  |  |  |  |
|  |  | 2.4.3 Communication difficulties with family, friends and colleagues |  |  |  |  |  |  |  |  |  |  |  |  |
|  |  | 2.5.4 Show sensitivity to strangers |  |  |  |  |  |  |  |  |  |  |  |  |
| 3. Physiological field | 3.1 Loop | 3.1.1 Edema |  | |  | | |  |  | |  | |  | |
|  |  | 3.1.2 Abnormal blood pressure |  |  |  |  |  |  |  |  |  |  |  |  |
|  |  | 3.1.3 Dyslipidemia |  |  |  |  |  |  |  |  |  |  |  |  |
|  |  | 3.1.4 Abnormal blood sugar |  |  |  |  |  |  |  |  |  |  |  |  |
|  | 3.2 Digestion - hydration | 3.2.1 Electrolyte Disturbance |  | |  | | |  |  | |  | |  | |
|  |  | 3.2.2 Loss of appetite |  | |  | | |  |  | |  | |  | |
|  |  | 3.2.3 Bad habits/vomiting/bloating |  | |  | | |  |  | |  | |  | |
|  | 3.3 Excretory function | 3.3.1 Diarrhea |  | |  | | |  |  | |  | |  | |
|  |  | 3.3.2 Constipation |  | |  | | |  |  | |  | |  | |
|  |  | 3.3.3 Frequent and urgent urination |  | |  | | |  |  | |  | |  | |
|  |  | 3.3.4 Abnormal urine volume |  | |  | | |  |  | |  | |  | |
|  | 3.4 Skin numbness | 3.4.1 Numbness in other parts of the skin |  | |  | | |  |  | |  | |  | |
|  |  | 3.4.2 Numbness in the skin scar at the surgical incision |  | |  | | |  |  | |  | |  | |
|  | 3.5 Infect | 3.5.1 Fungal infection |  | |  | | |  |  | |  | |  | |
|  |  | 3.5.2 Bacterial infection |  | |  | | |  |  | |  | |  | |
|  |  | 3.5.3 Other Infections |  | |  | | |  |  | |  | |  | |
|  | 3.6 Oral Hygiene | 3.6.1 Oral mucosal integrity |  | |  | | |  |  | |  | |  | |
|  |  | 3.6.2 Tooth decay |  | |  | | |  |  | |  | |  | |
|  |  | 3.6.3 Oral ulcers |  | |  | | |  |  | |  | |  | |
|  |  | 3.6.4 Pain/swelling/bleeding gums |  | |  | | |  |  | |  | |  | |
|  |  | 3.6.5 Gingivitis/Periodontitis |  | |  | | |  |  | |  | |  | |
| 4. Health behavior related fields | 4.1 Nutrition | 4.1.1 Daily body requirement |  | |  | |  | | |  | |  | |  |
|  |  | 4.1.2 Daily body intake |  | |  | |  | | |  | |  | |  |
|  |  | 4.1.3 BMI index |  | |  | |  | | |  | |  | |  |
|  | 4.2 Sleep and rest patterns | 4.2.1 Difficulty falling asleep |  | |  |  | | | |  | |  | |  |
|  |  | 4.2.2 Sitting up at night |  | |  |  | | | |  | |  | |  |
|  |  | 4.2.3 Insufficient sleep and rest |  | |  |  | | | |  | |  | |  |
|  |  | 4.2.4 Insomnia |  | |  |  | | | |  | |  | |  |
|  | 4.3 Physical Activity | 4.3.1 General physical activity without discomfort |  |  | |  | | | |  | |  | |  |
|  |  | 4.3.2 Feeling chest tightness and fatigue after general physical activity |  |  | |  | | | |  | |  | |  |
|  |  | 4.3.3 Discomfort such as chest tightness after mild activity |  |  | |  | | | |  | |  | |  |
|  |  | 4.3.4 Discomfort symptoms such as chest tightness still occur during rest |  |  | |  | | | |  | |  | |  |
|  |  | 4.3.5 Lack of exercise program |  |  | |  | | | |  | |  | |  |
|  | 4.4 Knowledge | 4.4.1 Describe postoperative dietary precautions |  |  | |  | | | |  | |  | |  |
|  |  | 4.4.2 Describe the precautions for postoperative rehabilitation exercise |  |  | |  | | | |  | |  | |  |
|  |  | 4.4.3 Describe the precautions for postoperative condition monitoring |  |  | |  | | | |  | |  | |  |
|  |  | 4.4.4 Describe the precautions for postoperative drug management |  |  | |  | | | |  | |  | |  |
|  | 4.5 Substance Abuse | 4.5.1 Alcoholism |  |  | |  | | | |  | |  | |  |
|  |  | 4.5.2 Smoking |  |  | |  | | | |  | |  | |  |
|  |  | 4.5.3 Other bad eating habits |  |  | |  | | | |  | |  | |  |
|  | 4.6 Drug treatment compliance | 4.6.1 Failure to take medication as prescribed |  |  | |  | | | |  | |  | |  |
|  |  | 4.6.2 Drug side effects occur |  |  | |  | | | |  | |  | |  |
|  |  | 4.6.3 Lack of drug expertise |  |  | |  | | | |  | |  | |  |
|  |  | 4.6.4 Improper storage of drugs |  |  | |  | | | |  | |  | |  |
|  | 4.7 Health Status Supervision | 4.7.1 Not receiving extended care |  |  | |  | | | |  | |  | |  |
|  |  | 4.7.2 Failure to follow up as requested |  |  | |  | | | |  | |  | |  |
|  |  | 4.7.3 Failure to return for examination as required |  |  | |  | | | |  | |  | |  |
